# Enzyme Linked Immuno-Spot; a Useful Tool in the Search for Elusive Immune Markers in Common Pediatric Immunological Diseases 

**DOI:** 10.3390/cells1020141

**Published:** 2012-05-29

**Authors:** Maria Faresjö

**Affiliations:** The Biomedical Platform, Department of Natural Science and Biomedicine, School of Health Sciences, Jönköping University and County Hospital, Ryhov, Jönköping S-551 11, Sweden; Email: maria.faresjo@hhj.hj.se; Tel.: +46-36-10-12-96

**Keywords:** Enzyme Linked Immuno-spot (ELISPOT), pediatric immunological diseases, immune markers, cytokines

## Abstract

In order to provide better therapy we strive to increase our knowledge of how the immune system behaves and communicates in common pediatric immunological diseases, such as type 1 diabetes, allergic and celiac diseases. However, when dealing with pediatric diseases, where study subjects are almost exclusively children, blood volumes available for immunological studies are limited and as such must be carefully handled and used to their full extent. Single immune markers can easily be detected by a traditional Enzyme Linked Immunosorbent Assay (ELISA), whereas multiple markers can be detected by a fluorochrome (Luminex) or electrochemiluminescence (MSD) technique. These techniques however are sometimes not sensitive enough to detect low levels of secreted immune markers in limited sample sizes. To detect immune markers at the single-cell level, an Enzyme Linked Immuno-spot (ELISPOT) can be used to pin-point elusive immune markers in common pediatric immunological diseases.

## 1. Introduction

Immune homeostasis should be controlled through the innate and adaptive systems used to maintain a balance between tolerance and suppression. Potentially dangerous lymphocytes can develop if self-reactive lymphocytes escape negative selection [1], or if a released self-antigen causes activation of peripheral autoreactive lymphocytes and subsequently results in the development of an autoimmune disease [2]. 

**Figure 1:**
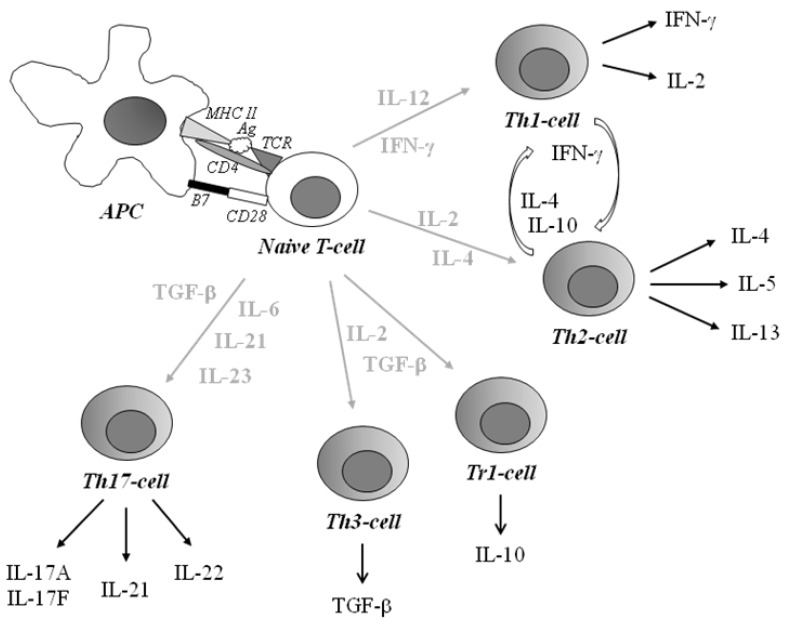
Schematic overview of T-helper subsets. The antigen presenting cell (APC) presents a degraded antigen (Ag) bound to the MHC class II. The complex is recognized by the T-cell receptor (TCR) and delivers a first signal for T-cell activation and a secondary signal by interaction between B7 and CD28. Through stimulation, a naïve T-cell can differentiate into different T-helper (Th) cells. Th1-cells, producing IFN- γ and IL-2, are associated with autoimmunity and intracellular pathogens whereas Th2-cells are associated with allergy and asthma and extracellular parasites and produce IL-4, -5 and -13 upon activation. Inducible antigen-specific sub-populations of CD4^+^ T-regulatory (Treg) cells are IL-10-producing T-regulatory cell type 1 (Tr1) cells and transforming growth factor (TGF)-β-secreting Th3 cells. Th17-cells producing IL-17A, -17F, -21 and IL-22 are capable of inducing inflammation and autoimmunity.

The local cytokine milieu is crucial for the differentiation of naïve T-helper (Th) cells. Immune responses driven by both Th1 and Th2 lymphocytes participate in the development of pathological cytotoxic conditions. In an attempt to classify activated Th-cells, they are grouped by their secretion of cytokines and to some extent by secretion of chemokines (Figure 1). A given cytokine might promote autoimmunity, or alternatively suppress the autoimmune and/or inflammatory process. Interferon (IFN)-γ and interleukin (IL)-2, produced by Th1-like lymphocytes, are thought to contribute to cell-mediated immunity, while humoral immunity has been suggested to be the main activity of Th2-like lymphocytes producing cytokines such as IL-4, -5 and IL-13 [3]. The Th2 lineage, known to secrete IL-4 and IL-13, induce IgE and IL-5 production which induces eosinophil growth and differentiation for the protection against parasites [4]. Th1-associated IFN-γ selectively inhibits proliferation of Th2-cells [5], whereas IL-4 and IL-10 inhibits cytokines synthesized by Th1-cells [6]. This cross-regulation may partly explain the strong biases towards Th1 or Th2 responses during several infections in mice and humans [7]. Inducible antigen-specific subset populations of CD4^+^ T-regulatory (Treg) cells are the IL-10-producing T-regulatory cell type 1 (Tr1) cells [8] and transforming growth factor (TGF)-β-secreting Th3 cells [9]. The main suppressive mechanism of Th3 cells is dependent on the production of TGF-β, which suppresses the proliferation of Th1 and Th2 cells.

An additional T-cell subset, distinct from IFN-γ-producing Th1 cells as well as Th2 cells, capable of inducing inflammation and autoimmunity, is named Th17 and produces IL-17A, -17F, -21 and IL-22 [10,11]. One hypothesis is that Th1- and Th17-cells might cooperate to induce the development of organ-specific autoimmunity, similarly as to in infectious diseases, in which IFN-γ and IL-17 might work together to increase resistance to infectious diseases [12]. It has also been described that IL-23 is one of the most potent inducers of these interleukins.

Type 1 diabetes, as well as allergic and celiac diseases are common immunological diseases in children. Cytokines form a complex network of interactions, suggesting a need to further understand how the immune system behaves and communicates in these diseases. 

Detection of single immune markers can be easily accomplished using a traditional Enzyme Linked Immunosorbent Assay (ELISA), whereas multiplex markers can be detected by a fluorochrome (Luminex) or electrochemiluminescence (MSD) technique. These techniques however are sometimes not sensitive enough to detect low levels of secreted immune markers in limited sample sizes. Due to difficulties in the detection of e.g., IL-4 [13], only limited amounts of human data exists.

An Enzyme Linked Immuno-spot (ELISPOT) permits the evaluation of a single antigen-specific memory T-cell with regard to frequency (clonal size) and cytokine signature. As such, an ELISPOT is considered advantageous for studies aimed at pin-pointing elusive immune markers in common pediatric immunological diseases.

## 2. Common Pediatric Immunological Diseases

### 2.1. Type 1 Diabetes

Type 1 diabetes (T1D), classified as an autoimmune disease, is known to be caused by the destruction of pancreatic beta-cells in genetically predisposed individuals [14,15]. The destruction is limited to the pancreatic islet beta-cells, resulting in absolute insulin deficiency and hyperglycemia. This autoimmune process is thought to exist for months or even years in a pre-clinical phase, with classic manifestation of the diseases, including hyperglycemia and ketosis, occurring only after most of the insulin-producing beta-cells have been destroyed [14,16]. 

Sweden has the second highest incidence rate of T1D in the world [17], with only Finland having more affected individuals [18]. These two Fennoscandian neighbours share high life standards and a high prevalence of risk-genes in the population.

#### Immunological Aspects of Type 1 Diabetes

Type 1 diabetes is suggested to be a result of the breakdown of mechanisms assuring the recognition of self and non-self that is a hallmark feature of autoimmune diseases. Th-lymphocytes, especially of a Th1-like subtype secreting dominantly interferon IFN-γ, IL-2 and TNF-β, have been shown to be important for the destruction of the insulin-producing beta-cells [14,19]. Pro-inflammatory cytokines, especially TNF-α and IL-6, and the chemokines monokine upregulated by IFN-γ (MIG), macrophage inflammatory protein (MIP), monocyte chemoattractant protein (MCP)-1 and IFN-γ-inducible protein 10 (IP-10) share a potent function of migration and have been shown to home inflammatory sites [20]. In contrast, Th2-like cytokines, e.g., IL-4, IL-5 and IL-13, have been shown to be downregulated during this organ-specific autoimmune process [21,22,23]. Thus, a dominant Th1-like profile before the clinical onset of T1D may be a major factor for the destruction of the insulin-producing beta-cells [22,23,24,25]. 

### 2.2. Allergic Disease

Allergy is the outcome of the immune system reacting towards harmless antigens (called allergens) that are normally tolerated. Typical allergic symptoms are asthma, rhinitis, conjunctivitis, eczema and gastrointestinal reactions. The so-called “atopic march” is the natural history of atopic manifestations—starting with atopic eczema and gastrointestinal problems in infancy being replaced by asthma and rhino-conjunctivitis, to inhalant allergens (birch pollen and pet allergens) often debuting in pre-school ages [26]. Accordingly, children with atopic eczema early in life are more prone to develop asthma and/or rhinitis than children in the general population [26]. 

During the past few decades the prevalence of allergic disease has increased dramatically in industrialized, high-income countries. Up to one third of all children have, or had, some allergic symptoms during childhood [27]. 

#### Immunological Aspects of Allergic Disease

Allergy is associated with Th2-like immunity to allergens in affected tissues [28]. Such patients have a hereditary predisposition to produce IgE antibodies against common environmental allergens. Th2 cells produce IgE synthesis stimulating IL-4 [4] and eosinophilia promoting IL-5 [29]. Typically, IgE antibodies to food allergens develop at the beginning of the “atopic march” accompanied by atopic eczema and urticaria, often succeeded by bronchial asthma and allergic rhinoconjunctivitis [30]. In contrast, IFN-γ from Th1 cells downregulate IgE synthesis [4]. 

### 2.3. Celiac Disease

Celiac disease is one of the most common chronic pediatric diseases in many countries. It is precipitated upon exposure to the dietary wheat gluten or its component gliadin in genetically susceptible subjects [31]. The disease is characterized by various degrees of crypt cell hyperplasia and villous atrophy which is manifested as diarrhea, flatulence, weight loss and fatigue. 

Three autoantibodies are usually seen in patients with celiac disease, antibodies against gliadin (AGA), endomysium (EMA) and tissue transglutaminse (tTGA) [32]. The enzyme tissue transglutaminse has been reported to modify the gliadin peptides via deamidation, thereby binding gliadin with higher affinity to HLA-DQ2 and HLA-DQ8, causing an epitope that is recognized by T-cells derived from the gut and increasing gliadin-specific reactivity of T-cells [33,34]. The disease heals and autoantibodies disappear after gluten withdrawal. 

#### Immunological Aspects of Celiac Disease

Celiac disease is believed to have a Th1-deviated immune response. Gluten appears to induce a non-proliferative activation of CD4+ lamina propria T-cells, especially activated Th1-like cells secreting IFN-γ [35]. Small intestinal biopsies from children with untreated celiac disease show pronounced Th1 gene expression together with increased expression of IL-2 [36,37,38]. In contrast, a percentage of samples from children with untreated celiac disease was found to have the same expression of IL-4 mRNA as controls [36,37]. However, one year after the introduction of a gluten-free diet, the transcription of IFN-γ was shown to be downregulated [38]. 

## 3. Immunological Methods Suitable for Detection of Cytokines

During an immune response low numbers of resting naïve or memory cells that do not secrete cytokines clonally expand and acquire a cytokine-expressing phenotype. Thus, cytokines play a central role in the enhancement and control of a developing immune response through their regulatory influence on Th cells, monocytes and other immune cells. However, cytokines act mainly in a paracrine and autocrine manner. In fact, cytokines are released and consumed locally at the site where the immune reaction occurs, and therefore most cytokines are detected at very low levels in peripheral blood. Detecting low levels of secreted immune markers in peripheral blood, especially in the limited sample size obtained from small children, is the challenge for the researcher. 

The gold standard for determining cytokine concentrations has long been the ***Enzyme Linked Immuno Sorbent Assay*** (ELISA). An ELISA allows cytokines and chemokines in body fluids, as well as in cell-culture supernatants of stimulated immune cells, to be detected. This technique generally has high specificity and is relatively easy to perform. The disadvantage is that only one single marker is detected in each assay.

To detect multiple markers, a multiplex ***fluorochrome technique***, Luminex^TM^, is used. This is a bead-based sandwich immunoassay that combines an ELISA with flow cytometry [39]. The technique uses individually dyed microbeads that have monoclonal antibodies directed against the cyto- and chemokines of interest and allows simultaneous detection of up to nearly 100 cyto- and chemokines in a dual laser flow analyser. The Luminex technique allows multiple cytokines to be measured simultaneously in small sample volumes and provides a convenient and sensitive tool for the detection of a large number of extracellular secreted cytokines to characterize cytokine profiles [40]. However, simultaneous detection of markers in one multiplex assay can sometimes be in conflict with the optimal sensitivity for each specific marker.

The ***electrochemiluminescence technique (MSD)*** is another multiplex immunoassay platform for the quantification of proteins in biological samples. In this assay a plate contains one to ten antibodies per well. The detection reagent is a compound called ruthenium (II) tris-bipyridine-(4-methylsulfonate) that chemiluminesces only upon electrical stimulation. This technique offers several advantages, including low background and usage of small sample-sizes.

## 4. Enzyme Linked Immuno-Spot (ELISPOT)

Enzyme Linked Immuno-spot (ELISPOT) is a technique by which immune markers, e.g., cytokine and chemokine secretion, can be detected at the single-cell level since secreted cytokines are captured and accumulated in the ELISPOT plate. ELISPOT was initially performed for detection of antigen-secreting cells [41] and later adapted for enumeration of cytokine-producing cells at the single-cell level [42]. The ELISPOT assay provides both qualitative (type of immune protein) and quantitative (number of responding cells) information, as each spot that develops in the assay represents a single reactive cell.

### 4.1. Methodological Principal

**Figure 2:**
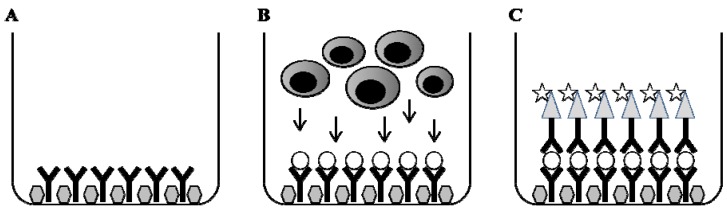
Methodological principal of ELISPOT. The wells of the ELISPOT-plate are coated with a primary antibody and thereafter non-specific binding sites are blocked (A). Cells are added in the presence or absence of specific stimulus. During incubation, cells become activated by the stimulus and start to produce and secrete, e.g., cytokine that binds to the capture antibody (B). Cells are removed and a detection antibody, which may be directly conjugated with enzyme or biotinylated, is added. Finally, a substrate will form a colored spot at the location of the secreting cell (C). By counting the number of spots in stimulated cultures and controls without stimulus the frequency of responding cells is determined.

In principal, the immune markers of interest, e.g., cytokines, are captured directly on the surface as a cytokine-antibody complex forming a spot around each cell secreting the cytokine of interest (Figure 2). The spot size and morphology reflects the kinetics and the quality of the cytokine production by individual cells over the entire test period.

### 4.2. The Use of ELISPOT in Search for Immune Markers in Pediatric Immunological Diseases

Enzyme Linked Immuno-spot permits evaluation of antigen-specific memory T-cells at the single-cell level with regard to frequency (clonal size) and cytokine signature. This makes a strong case for the use of ELISPOT for studies of immune markers, for example in common pediatric immunological diseases. 

Focusing on first-degree relatives of *type 1 diabetes* patients (islet antibody-positive) with very high risk of developing the disease, an overwhelming spontaneous secretion of IFN-γ has been detected in peripheral blood mononuclear cells measured by ELISPOT [22]. In addition, healthy high-risk individuals responded with an increased secretion of IL-4 from autoantigen-stimulation [22]. The ELISPOT technique was also able to distinguish immunological differences between those high-risk individuals that developed T1D and those remaining healthy [43]. In contrast, IFN-γ and IL-10 T-cell responses, studied by ELISPOT in first-degree relatives of T1D patients with very low risk (islet antibody-negative), showed balanced pro-inflammatory and regulatory T-cell response [44].

A low number of IL-12-producing cord blood mononuclear cells (CBMC) is associated with *IgE sensitization* during early childhood [45]. By ELISPOT-technique it has been shown that IgE-sensitized children, at two years of age, exhibited increased numbers of IL-4-producing cells in response to phytohaemagglutinin, as well as an increase in IL-10- and IL-12-producing cells when exposed to the allergen, ovalbumin [46]. In addition, TNF-α- or IL-10-producing peripheral blood mononuclear cells in children with atopic dermatitis with specific casein serum IgE has been detected by ELISPOT as being higher when compared to those children without this specific IgE response [47]. 

In children with untreated *celiac disease*, the numbers of IFN-γ-producing cells, detected by ELISPOT is shown to be increased and actually, after gluten challenge the numbers of IFN-γ-producing cells still remain high [48]. Interleukin-10 and IFN-γ ELISPOT assays for detection of gliadin specific T-cell lines indicates that recombinant human IL-10 abrogated the IFN-γ response to gliadin in patients with celiac disease [49]. This result indicates that recombinant human IL-10 induces a long term hyporesponsiveness of gliadin specific mucosal T-cells [49]. It has also been shown that an *in vivo* gluten challenge is a simple and safe method that allows relevant T-cells to be analysed and quantified in peripheral blood by ELISPOT [50]. 

The ELISPOT technique has also been used to characterize the Th1/Th2-like profile in children with combinations of the immunological diseases: *T1D, allergic and celiac diseases*. Children suffering from both T1D (Th1-prone) and allergy (Th2-prone) were found to react distinctly to general mitogen stimulation [51]. In contrast, children suffering from two Th1-dominated diseases (T1D and celiac disease) showed hardly any response to either food or inhalation allergens [51]. These results, achieved by the use of ELISPOT, indicate an important interplay between common immunological diseases in children [51].

Collectively, these results indicate that the ELISPOT technique is preferable for immunological studies focusing on detection of cytokines, e.g., IL-4, -10, -12 and IFN-γ, in children with immunological diseases.

### 4.3. Methodological Aspects of ELISPOT

By virtue of the exquisite sensitivity of the ELISPOT assay, frequency analysis of rare cell populations (e.g., antigen-specific responses) which were not possible before are now relatively easy. 

In a methodological study, three assays that can detect IL-4 responses; ELISPOT, ELISA and real-time reverse transcription polymerase chain reaction (RT-PCR) were compared in a cohort including atopic or nonatopic individuals, as well as pregnant women [52]. The authors claim that the ELISPOT assay displayed the highest sensitivity and was the only assay that could detect spontaneous secretion of IL-4 in all analysed samples [52]. Further, from comparing detection of IFN-γ by ELISPOT with intracytoplasmic immunofluorescence and ELISA from tetanus toxoid *in vitro* stimulation, it was shown that the ELISPOT assay could detect antigen-specific T-cells even in the absence of detectable IFN-γ in the culture supernatants [53].

A T-cell workshop comparing different cytokine ELISPOT assay formats demonstrated the potential for detection of low-level autoreactive T-cell responses for studies in T1D [54]. In a pre-testing of the workshop, the responses to islet antigen peptides by autoreactive T-cells demonstrated a very low frequency of responders cells (in the range of 1:100,000) and thus provided a great challenge to the sensitivity of the assay [54]. Even with clear differences between the assay performance within the workshop, the results presented demonstrated that low level recall antigen- and peptide specific auto-reactive T-cell responses were detectable by a cytokine enzyme ELISPOT and may be reproduced over time within the same assay format [54]. 

The limit of detection by ELISPOT is below 1/100,000, rendering enumeration of, e.g., elusive immune cells. The ELISPOT assay has also been proven to be up to 400 times more sensitive than an ELISA assay [55]. This exceptional sensitivity is in part due to the fact that the product is rapidly captured around the secreting cell: before it is either diluted in the supernatant, captured by receptors of adjacent cells, or degraded.

## 5. Concluding Remarks

By virtue of the exquisite sensitivity of the ELISPOT assay, frequency analysis of rare cell populations, which were not possible before, are now relatively easy. Most importantly, the ELISPOT technique has shown to be a sensitive and reliable immunological method for studies of immune markers at the single-cell level. Thus, the ELISPOT technique has proven useful as a tool in the search to pin-point elusive immune markers in common pediatric immunological diseases.
